# Validation of Core and Whole-Genome Multi-Locus Sequence Typing Schemes for Shiga-Toxin-Producing *E. coli* (STEC) Outbreak Detection in a National Surveillance Network, PulseNet 2.0, USA

**DOI:** 10.3390/microorganisms13061310

**Published:** 2025-06-04

**Authors:** Molly M. Leeper, Morgan N. Schroeder, Taylor Griswold, Mohit Thakur, Krittika Krishnan, Lee S. Katz, Kelley B. Hise, Grant M. Williams, Steven G. Stroika, Sung B. Im, Rebecca L. Lindsey, Peyton A. Smith, Jasmine Huffman, Alyssa Kelley, Sara Cleland, Alan J. Collins, Shruti Gautam, Eishita Tyagi, Subin Park, João A. Carriço, Miguel P. Machado, Hannes Pouseele, Dolf Michielsen, Heather A. Carleton

**Affiliations:** 1 Division of Foodborne, Waterborne, and Environmental Diseases, Centers for Disease Control and Prevention, Atlanta, GA 30329, USA; vgl0@cdc.gov (M.N.S.); tgriswold@cdc.gov (T.G.); pei6@cdc.gov (M.T.); uge1@cdc.gov (K.K.); gzu2@cdc.gov (L.S.K.); kpb6@cdc.gov (K.B.H.); fid4@cdc.gov (G.M.W.); fru3@cdc.gov (S.G.S.); wla9@cdc.gov (S.B.I.); koj1@cdc.gov (P.A.S.); mqx5@cdc.gov (J.H.); lsz0@cdc.gov (A.K.); nej1@cdc.gov (S.P.); 2 Applied Science Research and Technology, Inc., Smyrna, GA 30080, USA; 3 Booz Allen Hamilton, Atlanta, GA 30309, USA; pok2@cdc.gov (S.C.); tpz3@cdc.gov (S.G.); vjn0@cdc.gov (E.T.); 4 IHRC Inc., Atlanta, GA 30346, USA; 5 bioMérieux Portugal, 2795-197 Linda-a-Velha, Portugal; joaoandre.carrico@biomerieux.com (J.A.C.); miguelpaulo.machado@biomerieux.com (M.P.M.); 6 bioMérieux Benelux, 1030 Schaerbeek, Belgium; hannes.pouseele@biomerieux.com (H.P.); dolf.michielsen@biomerieux.com (D.M.)

**Keywords:** PulseNet 2.0, *Escherichia coli*, STEC, whole genome sequencing, outbreak, cgMLST, wgMLST, hqSNP

## Abstract

Shiga-toxin-producing *E. coli* (STEC) is a leading causing of bacterial foodborne and zoonotic illnesses in the USA. Whole-genome sequencing (WGS) is a powerful tool used in public health and microbiology for the detection, surveillance, and outbreak investigation of STEC. In this study, we applied three WGS-based subtyping methods, high quality single-nucleotide polymorphism (hqSNP) analysis, whole genome multi-locus sequence typing using chromosome-associated loci [wgMLST (chrom)], and core genome multi-locus sequence typing (cgMLST), to isolate sequences from 11 STEC outbreaks. For each outbreak, we evaluated the concordance between subtyping methods using pairwise genomic differences (number of SNPs or alleles), linear regression models, and tanglegrams. Pairwise genomic differences were highly concordant between methods for all but one outbreak, which was associated with international travel. The slopes of the regressions for hqSNP vs. allele differences were 0.432 (cgMLST) and 0.966 wgMLST (chrom); the slope was 1.914 for cgMLST vs. wgMLST (chrom) differences. Tanglegrams comprised of outbreak and sporadic sequences showed moderate clustering concordance between methods, where Baker’s Gamma Indices (BGIs) ranged between 0.35 and 0.99 and Cophenetic Correlation Coefficients (CCCs) were ≥0.88 across all outbreaks. The K-means analysis using the Silhouette method showed the clear separation of outbreak groups with average silhouette widths ≥0.87 across all methods. This study validates the use of cgMLST for the national surveillance of STEC illness clusters using the PulseNet 2.0 system and demonstrates that hqSNP or wgMLST can be used for further resolution.

## 1. Introduction

*Escherichia coli* (*E. coli*) is a large and diverse group of Gram-negative bacteria found in the environment and in the intestines of people and animals. While most strains of *E. coli* are harmless and are part of healthy intestinal tracts, some *E. coli* are pathogenic, with diarrheagenic strains causing symptoms such as stomach cramps, vomiting, fever, and watery or bloody diarrhea. Diarrheagenic *E. coli* are categorized into six pathotypes: Shiga-toxin-producing *E. coli* (STEC), Enterotoxigenic *E. coli* (ETEC), Enteropathogenic *E. coli* (EPEC), Enteroaggregative *E. coli* (EAEC), Diffusely Adherent *E. coli* (DAEC), and Enteroinvasive *E. coli* (EIEC), which produces a clinical manifestation similar to shigellosis [1]. Diarrheagenic *E. coli* is transmitted through the fecal–oral route via contaminated food or water, swimming in untreated water, or through direct contact with animals, people, or the environment [2].

One of these six pathotypes, STEC, causes human illness by producing a toxin known as Shiga toxin. STEC is a leading cause of foodborne and zoonotic illness in the United States, resulting in an estimated 265,000 illnesses, 2600 hospitalizations, and 30 deaths in the United States annually [3,4]. In some STEC infections, a condition known as hemolytic uremic syndrome (HUS) develops, which can cause anemia, acute renal failure, and death [5]. STEC bacteria are broadly categorized by serotype as STEC O157 and non-O157 STEC, and persons infected with STEC O157 strains are more likely to be hospitalized and develop HUS more frequently than those infected with non-O157 STEC strains [4,6]. While humans constitute the primary reservoir for non-STEC pathotypes, the intestinal tracts of animals, especially cattle and other ruminants, are the primary reservoirs of STEC [2].

Since 1996, PulseNet USA has served as the national molecular subtyping network for foodborne, waterborne, and one-health-related disease surveillance in the United States [7,8,9,10]. PulseNet USA is coordinated by the U.S. Centers for Disease Control and Prevention (CDC) and the Association of Public Health Laboratories (APHL) and comprises over 80 state and local public health laboratories and food regulatory federal agencies [10,11]. PulseNet-participating laboratories use standardized laboratory workflows and data-analysis tools to detect local and multistate foodborne and zoonotic illness clusters, including those caused by *E. coli*, primarily by STEC (both O157 and non-O157 serotypes) and EIEC/*Shigella* pathotypes. PulseNet surveillance also encompasses other *E. coli* pathotypes, such as EAEC, EPEC, and ETEC; however, these pathotypes are less frequently associated with human illness clusters in the United States. Additionally, with the exception of EIEC/*Shigella*, non-STEC pathotypes are not nationally notifiable [12]; thus, many states do not collect or report information on these pathotypes. Moreover, most clinical and public health laboratories do not use methods that can detect diarrheagenic *E. coli* from pathotypes other than STEC in stool samples [2].

Between 1996 and 2019, pulsed-field gel electrophoresis (PFGE) was the subtyping method used by PulseNet to detect clusters of foodborne illness. While PFGE has a long history of utility in enteric disease surveillance, whole-genome sequencing (WGS) offers improved discriminatory power and concordance with epidemiologic data when compared with PFGE [10,11,12,13]. In 2012, PulseNet began exploring opportunities for replacing PFGE with WGS, and in July 2019, PulseNet fully transitioned all enteric bacterial surveillance from PFGE to WGS. This included incorporating WGS data into PulseNet’s previously existing bioinformatics and information technology infrastructure, formerly a customized version of BioNumerics v7.6 software [14].

**Figure 1:**
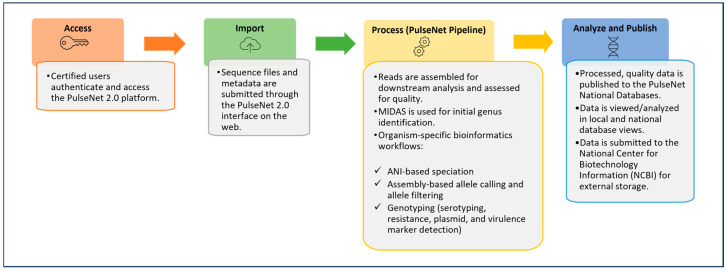
PulseNet 2.0 data analysis workflow.

The incorporation of WGS data within the PulseNet BioNumerics v7.6 databases presented challenges that necessitated frequent updates and customizations within the software. These challenges and the need to share all bioinformatics tools as open source brought about the development of a new bioinformatics and data management system for the PulseNet USA network. In September 2024, PulseNet transitioned to a new cloud-based, modular, open-source platform, referred to as PulseNet 2.0. Data analysis within PulseNet 2.0 follows a standardized workflow that performs sequence quality assessments, the de novo assembly of sequenced genomes, speciation, sequence quality assessments, allele calling, and various genotyping tasks, such as serotyping, resistance profiling, and the identification of virulence markers and plasmids (Figure 1), using Nextflow v25.04.2 https://github.com/nextflow-io/nextflow as a workflow manager (last accessed for this study on 1 April 2025). The PulseNet 2.0 system was designed for end-to-end data analysis, making it suitable for use with WGS, a method that permits multiple characterizations of isolate genomes using one workflow [10,15,16,17].

**Figure 2:**
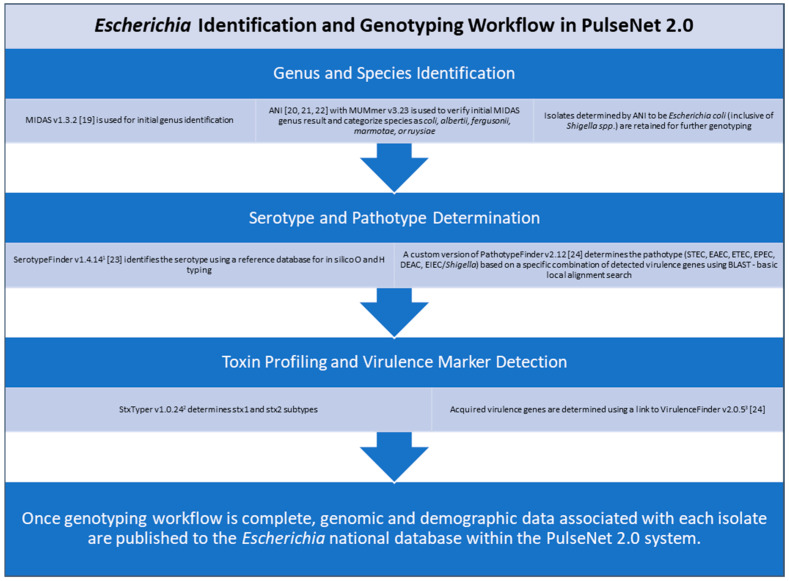
Overview of *Escherichia* identification and genotyping workflow in PulseNet 2.0. ^1^ https://cge.food.dtu.dk/services/SerotypeFinder/; ^2^ https://github.com/ncbi/stxtyper; ^3^ https://cge.food.dtu.dk/services/VirulenceFinder/. All websites were last accessed on 1 April 2025 for this study.

Specifically, for *E. coli*, some of these characterizations include species and serotype identification, pathotype determination, toxin profiling, and virulence marker detection [18,19,20,21,22,23,24]. Once the identification and genotyping workflow is complete, genomic and demographic data associated with each isolate are published in real time to the *Escherichia* national database within the PulseNet 2.0 system (Figure 2). The national database provides a centralized view of genomic and demographic data, allowing PulseNet data analysts at the CDC to rapidly detect multistate *Escherichia* illness clusters that have the potential to evolve into widespread outbreaks. For STEC (both O157 and non-O157), PulseNet’s primary national cluster definition is five or more clinical cases published to the PulseNet 2.0 national database within 60 days of each other and with cases relating to each other within 0 to10 allelic differences based on cgMLST [18].

As next-generation sequence technology has advanced, public health surveillance networks, such as PulseNet, have used various approaches for analyzing WGS data for foodborne disease surveillance. These include high-quality single-nucleotide polymorphism (hqSNP) analysis, core genome multi-locus sequence typing (cgMLST), and whole genome multi-locus sequence typing (wgMLST). hqSNP analyses compare isolate genomes to a closely related reference sequence to examine single-nucleotide changes in the DNA sequence. cgMLST analyses examine differences in the loci found in 95–98% of the reference strains used to build the allele scheme, and wgMLST analyses examine differences in either all loci or chromosomal loci found in the reference strains used to develop the allele schemes. These methods enable phylogenetic comparisons between sequenced isolates and are used to identify isolates that may have a common source within a foodborne or zoonotic outbreak [11,17,25,26,27]. While MLST-based methods require the up-front establishment of allele schema and methods, they are particularly suited for large-scale surveillance for their standardization and comparability, especially for epidemiological studies. On the other hand, hqSNP-based methods are often more powerful for the fine-scale resolution of genetic differences but can be more resource intensive and require the selection of an appropriate reference genome.

Previous studies have shown that unsupervised machine learning techniques can be used to cluster genomic data [28,29,30]. For example, K-means analysis, which divides a dataset into a predefined number of clusters (“K”), where data points within a cluster are more similar to each other than to points in other clusters [31], can be used to examine the phylogenetic clustering of isolate genomes. This clustering of data can be performed independently of an established, predefined cluster-detection threshold, making it an objective and external approach to enhance the validation of genomic-cluster-detection methods [28,30]. K-means clustering analysis has been successfully applied to research in various fields of biological science, such as clustering gene expression data or protein sequence data [32].

This study has two primary objectives: 1) to evaluate the overall concordance of three WGS-based subtyping methods: cgMLST, wgMLST using chromosome-associated loci [wgMLST (chrom)], and hqSNP for STEC human illness cluster detection in the United States, and 2) to evaluate the allele schemes built into the PulseNet 2.0 *Escherichia* national database to assess the reliability in detecting STEC outbreak clusters relative to hqSNP, a gold standard genomic comparison method. Multiple methods were used to meet these objectives, including an assessment of pairwise genomic differences across subtyping methods, linear regression models, phylogenetic clustering comparisons, and K-means clustering analysis. The findings of this study can be used to validate the use of allele-based methods for STEC illness cluster detection within the PulseNet 2.0 national USA surveillance network.

## 2. Materials and Methods

Selection of Isolate Datasets. A total of 251 STEC O157 and STEC non-O157 isolates from 10 foodborne and 1 travel-associated outbreak was selected from the PulseNet national database. Outbreaks occurred between 2016 and 2022 and had well-characterized sources based on epidemiologic investigations. Each outbreak was assigned a number between 01 and 11 for the study (Table 1). A total of 46 sporadic/non-outbreak STEC isolates was also selected from the PulseNet national database to evaluate the ability of the allele schemes to differentiate outbreak and sporadic/non-outbreak isolate sequences. Sporadic isolates were matched to individual outbreaks by serotype, as determined via traditional serotyping or WGS, and the selection of sporadic isolates was limited to those having collection dates within 6 months of the outbreak’s median collection date. Isolates were considered sporadic if they were not associated with any previously identified or investigated illness clusters. The number of sporadic isolates compared per outbreak ranged between 2 and 8 (Table 1). Raw sequence data files for the 297 isolates included in this study have been deposited in the National Center for Biotechnology (NCBI) Sequence Read Archive (SRA) under Bioproject PRJNA218110 (PulseNet *Escherichia coli* and *Shigella* genome sequencing), and accession numbers are listed in Supplementary Table S1.

Whole Genome Sequencing. WGS data were available for all 297 isolates in the study. Sequencing was performed on Illumina instruments using the Illumina Nextera XT or DNA Prep library preparation kits (San Diego, CA, USA) by PulseNet-participating public health laboratories or CDC according to the protocols available at https://www.aphl.org/programs/global_health/Pages/PulseNet-International-SOPs.aspx, last accessed for this study on 1 April 2025 [33].

**Figure 3:**
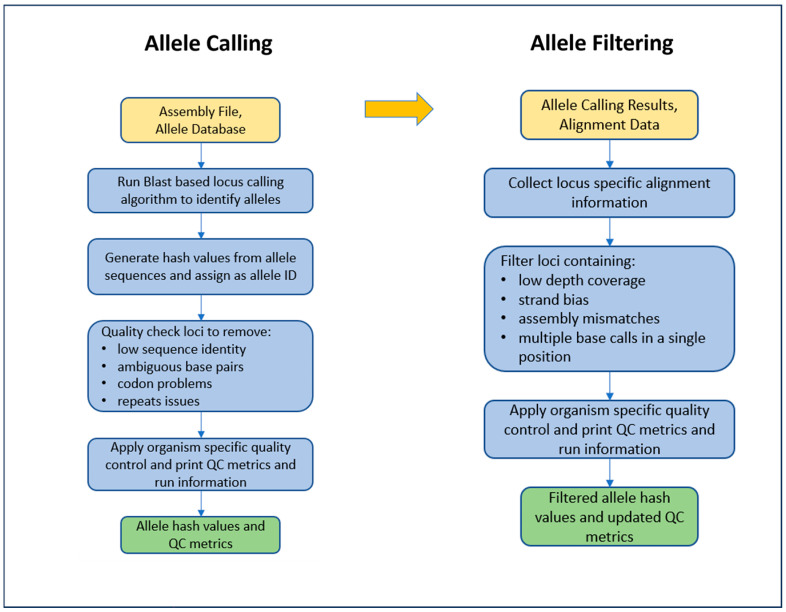
PulseNet 2.0 allele calling workflow.

WGS Analysis and Allele Calling. Illumina sequence read files for all isolates were linked and analyzed in the PulseNet 2.0 system. Genus identification was performed using MIDAS (v1.3.2) [19]. The raw reads were trimmed with fastp https://github.com/OpenGene/fastp (v0.32.2), last accessed for this study on 1 April 2025), using an average quality threshold of 30 and downsampled to 100× coverage with seqtk https://github.com/lh3/seqtk/releases (v1.3), last accessed for this study on 1 April 2025, using an expected genome size of 4.2 Mb. MIDAS (v1.3.2) was run a second time using the cleaned reads to detect any contamination. De novo assembly of the cleaned reads was performed with SPAdes (v3.15.5) [34] using the—isolate option. The cleaned reads were aligned back to the assembled genomes using BWA (Burrow–Wheeler Aligner) https://github.com/lh3/bwa (v0.7.17), last accessed for this study on 1 April 2025. The assembly was corrected by removing any contigs shorter than 500 bp, those with an average read depth below either a threshold of 15× or 25% of the assembly average depth, whichever value was greater, and those with a GC content less than 5%, as measured based on Samtools https://github.com/samtools (v1.16.1), last accessed for this study on 1 April 2025. The cleaned reads were mapped back to the corrected assembly to create cleaned BAM—Binary Alignment Map—files. The genus and species were identified using ANI with MUMmer https://github.com/chienchi/MUMmer (v3.23), last accessed for this study on 1 April 2025, and the genus result was verified with the MIDAS result. Sequences determined by ANI to be *E. coli* (including *Shigella* spp.) were retained for further analysis. Allele calls were generated using the PulseNet 2.0 MLST caller with the following steps: the corrected assemblies were compared against reference allele sequences in the PulseNet 2.0 MLST database repository https://github.com/ncezid-biome/pn2.0-mlst-databases (schema further described below under the heading “PulseNet 2.0 *Escherichia* Allele Schema”) using a BLASTn https://ncbiinsights.ncbi.nlm.nih.gov/2021/07/09/blast-2-12-0/ (v2.12), last accessed for this study on 1 April 2025), approach to find the presence of each locus. The query allele sequence was defined by the presence of start and stop codons (without nonsense mutations) and a minimum similarity of 85% against a reference allele. Loci that were likely repeated (fully or partially) elsewhere in the genome were ignored. The query sequences were hashed using the 64-bit MD5 algorithm and then transformed into a 56-bit integer https://github.com/ncezid-biome/pn2.0-mlst-databases?tab=readme-ov-file#hashing-function, last accessed for this study on 1 April 2025. A further filtration was performed using the aligned reads, and alleles that did not meet these minimum quality standards for each nucleotide call were removed: 65% homozygosity rate, depth of coverage greater than 5× depth, at least 1% of reads supporting each forward and reverse strand (Figure 3).

Sequence Quality Assessment. Once allele calling was performed, sequence quality was assessed for each genome. Genomes with <85% of alleles called within the cgMLST scheme were considered to fail quality. Genomes with ≥85% core alleles present were considered to pass quality if they met the following additional quality metric cut-offs: average coverage ≥40×; average base quality score ≥30; and assembly length = 4.2–5.9 Mbp. PulseNet uses an 85% core-allele-call threshold primarily to ensure data quality, consistency, and reliability in WGS-based subtyping across the network. Furthermore, when the additional quality metric cut-offs for the coverage, q-score, and length are met, sequences are more likely to meet the 85% cgMLST allele calling threshold, filtering out low-quality genomes from surveillance [11]. For sequences that passed quality, genotyping was performed (Figure 2) to determine the serotype, pathotype, and Shiga toxin profile (if applicable) of each isolate. Once genotyping was complete, sequences were published to the PulseNet 2.0 national database and submitted to NCBI’s sequence read archive (SRA) under Bioproject PRJNA218110.

High-Quality SNP Analysis. For hqSNP comparisons, CDC uses an hqSNP pipeline called Lyve-SET [25] to assess the phylogeny of isolates within an outbreak. The design of Lyve-SET was optimized for epidemiologic investigations and has shown that as the phylogenetic relatedness between isolate sequences increases, the likelihood of epidemiological relatedness increases [11,13,17,25,35,36]. High-quality SNP (hqSNP) data were generated for all outbreak and sporadic isolates included in the study. The hqSNP analyses were generated through Lyve-SET v1.1.4f with the default modules selected for mapping and SNP calling. Reads were cleaned during the preparation phase before running Lyve-SET. Prior to SNP calling, options were set according to the *Escherichia*-specific thresholds specified under the “*escherichia_coli*” configuration; Lyve-SET workflow option “—presets”, respectively [25]. An internal draft reference, belonging to the specified outbreak, or an external closed reference, neither associated with the outbreak or sporadic isolate set, was selected (Supplementary Table S2). Reference sequences were assembled using SPAdes v3.14.0 [34], and plasmids were masked on the generated SPAdes assemblies through identification and exclusion using PlasFlow v1.1 [37]. Phages were masked using the Lyve-SET workflow for all outbreaks by default. For each outbreak, two hqSNP analyses were performed where one contained solely outbreak-associated genomes and the second included the sporadic set for the outbreak. A phylogenetic tree (RaxML) [38] and pairwise SNP difference matrix were generated for each hqSNP analysis.

**Figure 4:**
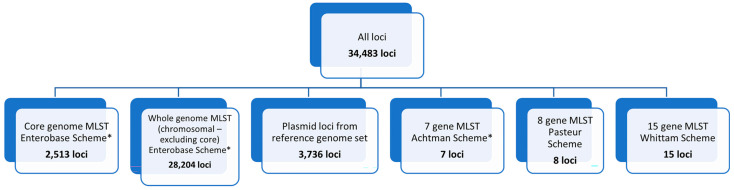
PulseNet 2.0 *Escherichia* schema. Number of loci included within schemes are shown for the overall scheme (all loci), core genome, whole genome (excluding core), plasmid, and 7-gene, 8-gene, and 15-gene MLST schemes. * indicates that the scheme is hosted on Enterobase: https://enterobase.warwick.ac.uk/ (last accessed for this study on 1 April 2025).

PulseNet 2.0 *Escherichia* Allele Schema. Currently, the PulseNet USA network uses allele-based methods for detecting *Escherichia* illness clusters, including those caused by STEC. Three MLST schemes have been incorporated into the PulseNet 2.0 national database for *Escherichia*. The core (cgMLST) scheme contains 2513 loci and represents the genes found in 95% or more of the reference strains used to develop the database [39]. The core and chromosomal accessory genes make up the whole-genome MLST (chromosomal) scheme, wgMLST (chrom), which contains 30,717 chromosomal loci, inclusive of the 2513 core genome loci. The wgMLST (all loci) scheme contains 34,483 loci and is inclusive of the core and accessory genome, as well as 3737 plasmid loci and loci from 7-gene, 8-gene, and 15-gene MLST schemes that are not already part of the core scheme (Figure 4). Locus names for all schemes incorporated into the PulseNet 2.0 national *Escherichia* database for are included in Supplementary Table S3.

Comparison of WGS-Based Subtyping Methods. The concordance between SNP- and allele-based methods was determined using multiple approaches, including pairwise genomic differences, linear regression models, phylogenetic tanglegrams, and K-means analysis. To evaluate pairwise genomic differences for the cgMLST and wgMLST (chrom) allele-based methods, allele differences between isolate genomes were converted into pairwise matrices for each outbreak within the PulseNet 2.0 system. Similarly, using hqSNP data generated for each outbreak, SNP differences between isolate genomes were determined and converted into pairwise matrices. Pairwise cg/wgMLST and hqSNP differences were combined into one overall profile per subtyping method, and a Pearson correlation coefficient was calculated in R Studio v1.4.1717 (Performance Analytics package), last accessed for this study on 01 January 2025, to show the overall correlation between each method, supported by a 95% confidence interval (CI). Appendix A describes a further comparison of pairwise differences obtained from PulseNet USA’s previous data management system, BioNumerics v.7.6.3, to those obtained from PulseNet 2.0.

For the linear regression models, three scatterplots were generated to compare the genomic differences produced by one subtyping method to that of the other two. Pairwise cgMLST and wgMLST (chrom) allele differences were plotted against their corresponding pairwise hqSNP differences, as well as to each other. For each scatterplot, a linear regression line was added to model the relationship between methods. Slopes of regression formulas, supported by 95% confidence intervals, were indicative of the genomic differences between subtyping methods among pairwise isolates. Y-intercepts were indicative of the pairwise cg/wgMLST (chrom) allele differences when hqSNP differences were zero or of the pairwise wgMLST (chrom) allele differences when cgMLST allele differences were zero. R^2^ values were used to determine how well the data fit each regression model (goodness of fit).

Tanglegrams (side-by-side facing dendrograms) were constructed to compare the phylogenies generated using each subtyping method [(cgMLST, wgMLST (chrom), and hqSNP)] when outbreak isolate sequences were combined with their corresponding sporadic/non-outbreak isolate sequences. Allele-based dendrograms were constructed in the PulseNet 2.0 system using absolute allelic differences. SNP-based dendrograms were constructed using the maximum likelihood method [40]. All allele and SNP-based dendrograms were converted to Newick format and assembled into tanglegrams in Base R v4.1.2 (dendextend package) [41], last accessed for this study on 01 November 2024, and the layout was optimized to minimize entanglement, as intricately tangled trees can become difficult to analyze, using the step2side method [42]. The statistical association of the branches in the two facing dendrograms was assessed using two measures: the Baker’s Gamma Index (BGI) and Cophenetic Correlation Coefficient (CCC). The Baker’s Gamma Index, also known as the Goodman–Kruskal–gamma index, is a statistical measure of the similarity between two hierarchical clustering trees and ranges from −1 to 1, with values closer to 1 indicating greater statistical similarity [43]. A value near 0 indicates that the trees are not statistically similar, and a negative value suggests strong disagreement between the dendrograms. The Cophenetic Correlation Coefficient is a statistical measurement that evaluates how well a dendrogram preserves the original distances between data points and is particularly useful for understanding clustering quality. A coefficient close to 1 indicates that the clustering algorithm preserves the original data structure well, while a lower coefficient suggests the clustering less accurately represents the distances [44]. Both measures were acquired using the *dendextend* package in Base R v4.1.2 [41].

For further validation, an unsupervised machine learning method, K-means analysis, was performed to compare the clustering results of the three WGS-based subtyping workflows. The Silhouette method [45] was applied in R/R Studio v1.4.1717 (Nbclust package) [46], last accessed for this study on 01 November 2024, to each dataset of combined outbreak and sporadic isolate sequences. This method identifies the optimal or most favorable number of clusters, or “K”, within each dataset. The optimal K value was based on the maximum Silhouette score, and a Pearson gamma coefficient (Km_stats function) expressed the statistical significance of the chosen K. In addition, for every outbreak, average silhouette widths were obtained for the outbreak isolate group (K1) and the sporadic isolate group (K2) (Km_stats function). For visualization, the dendrogram function (scikit-learn package v1.6.1) [47], last accessed for this study on 01 November 2024, in Python v3.9.21 Jupyter v1.1.1 notebooks [47] was used to perform a hierarchical divisive cluster analysis using single linkage, whereby single linkage considers the distance between clusters as the minimum distance, depicting the partitioning of K1 and K2 groups for each outbreak. This exercise was performed for each subtyping workflow using pairwise differences generated for each combined set of outbreak and sporadic isolate sequences.

## 3. Results

### 3.1. Summary of Outbreak Information

Table 1 (shown above under the heading “Selection of Isolate Datasets”) provides a summary of the 11 outbreaks included in this study. Six unique serotypes were represented among the outbreaks, including *E. coli* O157:H7 (4 outbreaks), *E. coli* O121:H19 (2 outbreaks), *E. coli* O26:H11 (2 outbreaks), *E. coli* O103:H2 (1 outbreak), *E. coli* O111:H8 (1 outbreak), and *E. coli* O5:H9 (1 outbreak). Collection dates ranged from 1 February 2016 to 7 July 2022, and all outbreaks were foodborne with the exception of outbreak 07, an *E. coli* O111:H8 outbreak associated with international travel.

### 3.2. Pairwise Genomic Differences

The range of SNP- and allele-based pairwise genomic differences between isolates is shown in Table 2 for each outbreak. For 10/11 outbreaks, SNP differences were mostly concordant with cgMLST and wgMLST (chrom) allele differences, differing by no more than 5 SNPs from the allele-based results. However, for outbreak 07, a travel-associated outbreak, SNP differences (0–19) were more closely aligned with wgMLST (chrom) differences (0–16) than to cgMLST allele differences (0–8) (Table 2).

### 3.3. Linear Regression Models

**Figure 5:**
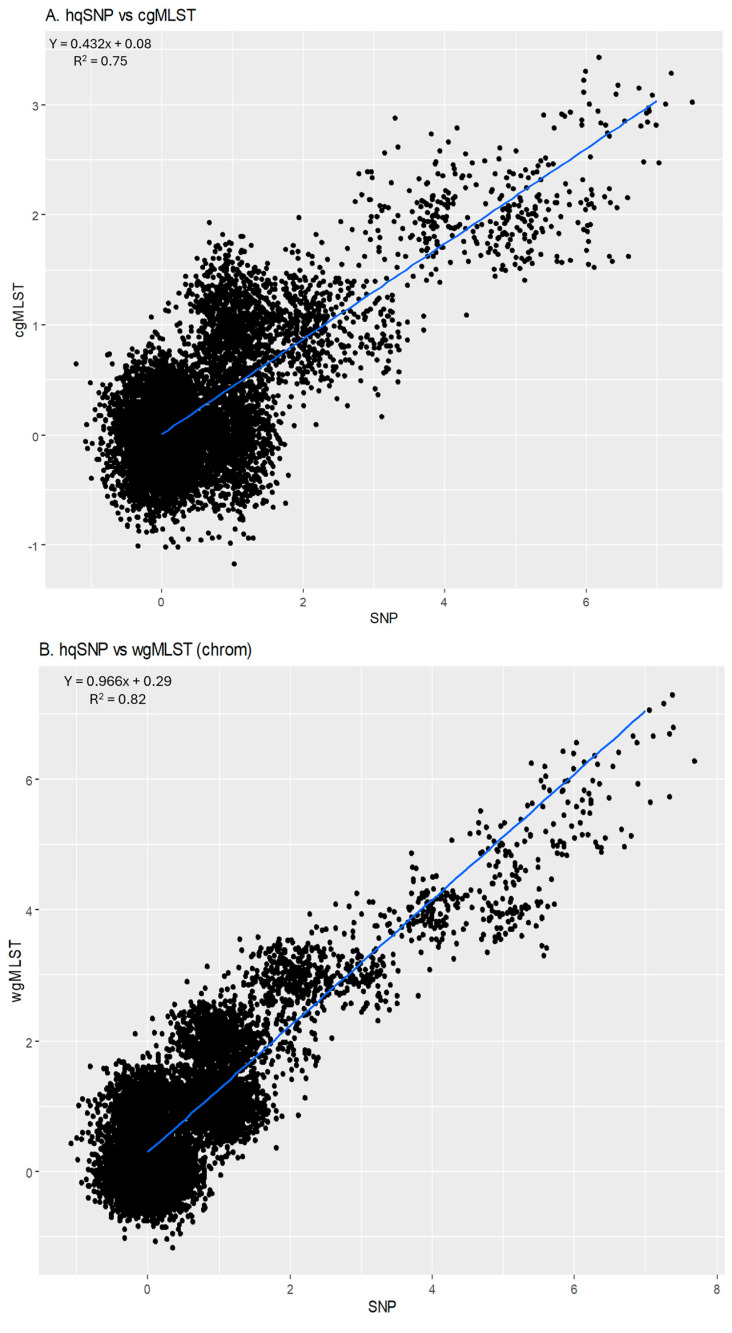

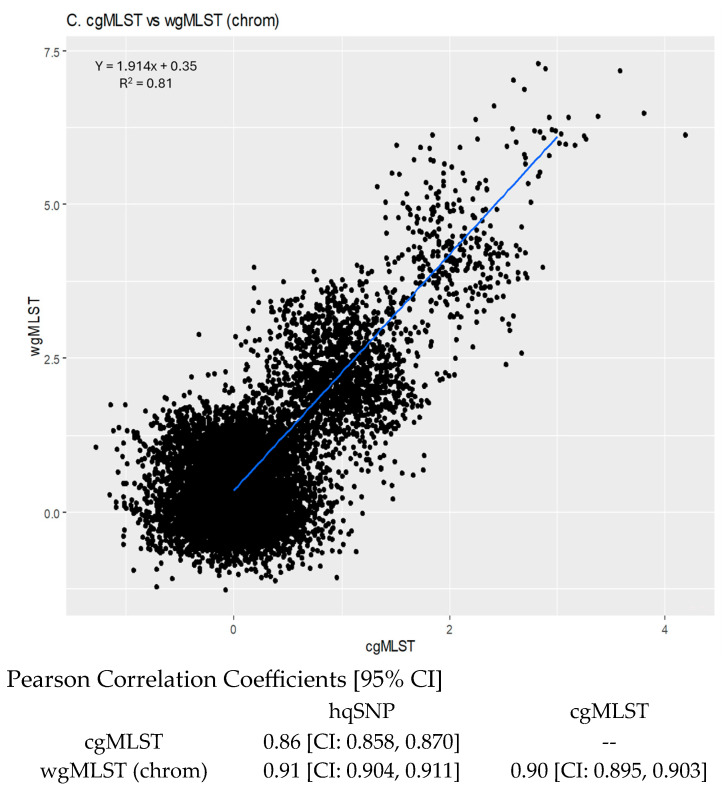
(**A**) Scatterplot of hqSNP differences vs. cgMLST differences. (**B**) Scatterplot of hqSNP differences vs. wgMLST (chrom) differences. (**C**) Scatterplot of cgMLST vs. wgMLST (chrom) differences. Regression equations and R^2^ values are displayed on the plots. Pearson correlation coefficients for each combination of pairwise matrices are shown below plots.

For all outbreak isolate sequences, allele-based cgMLST and wgMLST (chrom) pairwise genetic differences were plotted against their respective SNP differences and are shown in Figure 5A (cgMLST) and Figure 5B [(wgMLST (chrom)]. The slope of the linear regression for cgMLST vs. SNP pairwise differences was 0.432 [95% CI: 0.426, 0.437], indicating that there were lower cgMLST differences compared to SNP differences. The y-intercept comparing cgMLST allele differences to SNP differences was 0.08, indicating that sequences that were zero SNPs different were also close to zero cgMLST alleles different on average. The slope of the linear regression for wgMLST vs. SNP pairwise differences was 0.966 [95% CI: 0.956, 0.975], indicating that there were slightly lower wgMLST allele differences between pairwise isolates compared to SNP differences. The y-intercept comparing wgMLST (chrom) allele differences to SNP differences was 0.29, illustrating that sequences that were zero SNPs different were also <1 alleles different on average. The goodness of fit for these models, as measured based on an R^2^ value, was 0.75 for cgMLST vs. SNP and 0.82 for wgMLST (chrom) vs. SNP, reflecting moderate amounts of variation within the models. Outbreak 07 was removed from linear regression models due to outlying allele and SNP differences compared to other outbreaks.

For cgMLST vs. wgMLST (chrom), the slope of the linear regression was 1.914 [95% CI: 1.895, 1.933]; (R^2^ = 0.81), indicating that there were higher wgMLST (chrom) allele differences per cgMLST allele difference, as expected, since wgMLST incorporates more loci than cgMLST. The y-intercept was 0.35, illustrating that sequences that were zero cgMLST alleles different were less than 1 wgMLST (chrom) allele different (Figure 5C).

There was high correlation between methods when overall pairwise differences were compared, as indicated by Pearson correlation coefficients supported by 95% confidence intervals. For cgMLST vs. SNP, the correlation coefficient was 0.86 [CI: 0.858, 0.870]. For wgMLST (chrom) vs. SNP, the correlation coefficient was 0.91 [CI: 0.904, 0.911], and for cgMLST vs. wgMLST (chrom), the correlation coefficient was 0.90 [CI: 0.895, 0.903] (Figure 5).

### 3.4. Tanglegrams

**Figure 6:**
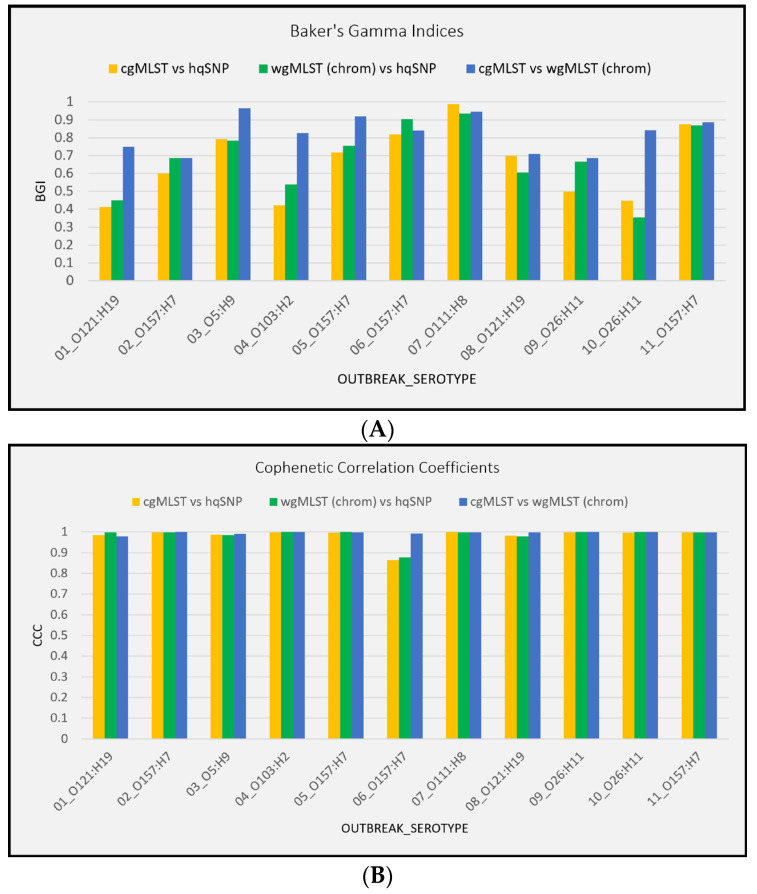
(**A**) Baker’s gamma indices for outbreak tanglegrams. (**B**) Cophenetic Correlation Coefficients for outbreak tanglegrams.

**Figure 7:**
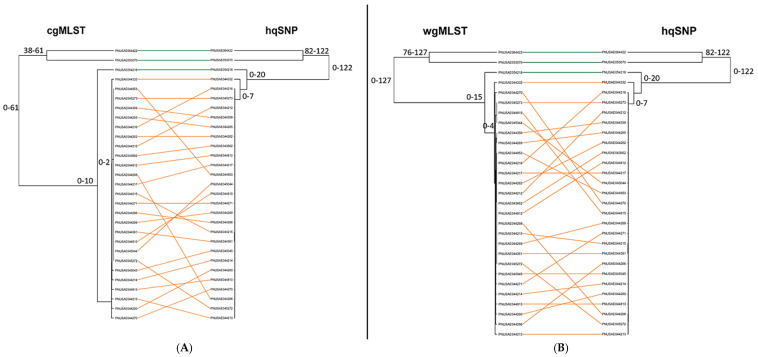
(**A**) Tanglegram of cgMLST and hqSNP clustering using single linkage for one representative outbreak (outbreak 04) and its corresponding sporadic/non-outbreak isolates. (**B**) Tanglegram of wgMLST (chrom) and hqSNP clustering for the same set of isolate sequences. Outbreak isolates are depicted in orange, and sporadic isolates are depicted in green. The tanglegram links tips with the same label to each other via a straight line. Allele/hqSNP differences are labeled at each node.

For all 11 outbreaks, tanglegrams showed moderate-to-high concordance between subtyping methods in terms of each method’s ability to separate outbreak and sporadic isolate sequences. For allele vs. hqSNP tanglegrams, BGI values ranged from 0.413 to 0.987 (cgMLST) and 0.354 to 0.936 [wgMLST (chrom)]. BGI values ranged from 0.686 to 0.964 when cgMLST was compared to wgMLST (chrom), representing statistically similar clustering between trees (Figure 6A, Supplementary Table S4). Across all three subtyping methods and for all 11 outbreaks, the Cophenetic Correlation Coefficient was ≥0.865, indicating the high fidelity of original pairwise distances in the dendrograms (Figure 6B, Supplementary Table S4). A visual representation of tanglegrams generated for one outbreak is shown in Figure 7.

### 3.5. K-Means Analysis

Across all three subtyping methods and for all 11 outbreaks, the Silhouette score was maximized at K = 2, designating 2 as the ideal number of clusters/groups within each combined set of outbreak and sporadic isolate sequences. The statistical significance of K = 2 was measured based on a Pearson gamma coefficient, which ranged from 0.85 to 0.99 across outbreaks, signifying robust clustering performance via the K-means analysis (Supplementary Table S5). For every outbreak, the average silhouette width for the outbreak isolate group was consistently high across subtyping methods and ranged from 0.92 to 0.99 (cgMLST), 0.89 to 0.99 [wgMLST (chrom)], and 0.87 to 0.99 (hqSNP), where a value close to 1.00 indicates more solid and cohesive clustering within groups. The average silhouette widths for the sporadic isolate groups showed more variation, as expected, since sporadic groups were not epidemiologically linked, ranging from 0.34 to 0.93 (cgMLST), 0.32 to 0.92 [wgMLST (chrom)], and 0.35 to 0.91 (hqSNP) (Supplementary Table S6). For all 11 outbreaks, a hierarchical divisive cluster analysis using single linkage showed consistent division of outbreak and sporadic isolates into two distinct groups across subtyping methods, as shown with cluster dendrograms. For 8 of the 11 outbreaks, K-means analysis assigned all outbreak and sporadic isolates into the correct groups based on ground truth data. For 3 of the 11 outbreaks (outbreaks 04, 06, and 11), K-means analysis incorrectly assigned between one and six isolates into the wrong group (either outbreak or sporadic) based on ground truth data. These incorrect assignments occurred across all three subtyping methods for these outbreaks, and except for outbreak 06, the same isolate(s) was/were incorrectly assigned to the wrong group across subtyping methods. (For outbreak 06, the same two sporadic isolates, PNUSAE026825 and PNUSAE033271, were incorrectly assigned to the outbreak group using cgMLST and wgMLST (chrom), but when using hqSNP, all six sporadic isolates were incorrectly assigned to the outbreak group). Cluster dendrograms are shown for all outbreaks in Supplementary Table S7A. Supplementary Table S7B lists the incorrectly assigned isolates for outbreaks 04, 06, and 11, as well as the minimum pairwise difference between each isolate and the incorrect group to which it was assigned.

### 3.6. Summary of Metrics

All analysis metrics obtained in this study are shown in Table 3, Table 4 and Table 5.

## 4. Discussion

As WGS continues to be used to support epidemiological investigations of foodborne and zoonotic outbreaks, validation of the methods used for routine cluster surveillance is essential. While allele-based cluster detection methods have been previously validated for other foodborne pathogens under PulseNet USA surveillance, namely *Campylobacter* and *Salmonella* [11,30], this study aimed to support the use of PulseNet’s allele-based cluster detection methods for STEC. This study demonstrated that the allele schemes (core genome and whole-genome using chromosomal loci) integrated into the PulseNet 2.0 *Escherichia* national database generate outputs that are highly concordant with the hqSNP analysis and well-aligned with epidemiological data. These findings establish allele-based methods as a reliable mechanism for STEC illness cluster detection in the United States using the PulseNet 2.0 system.

This study is one of the first to compare hqSNP and allele-based outputs using the PulseNet 2.0 allele calling workflow. Specific improvements in the PulseNet 2.0 allele calling workflow include (1) the incorporation of hash values for allele identification, (2) an allele-filtering step, which ensures high quality locus classification, leading to a more accurate analysis for outbreak detection, and (3) improved time and memory performance of allele calling and filtering. Overall, cloud-based data-processing and analysis pipelines, such as PulseNet 2.0, support scalable and shareable bioinformatics tools across multiple users, making PulseNet 2.0 more cost-effective, particularly in terms of the cost savings from faster, more accurate outbreak detection and responses. These improvements, coupled with the results of this study, should grant public health researchers assurance in using the PulseNet 2.0 system for STEC outbreak detection.

Using 11 well-characterized STEC outbreaks, this study showed concordance between subtyping methods when pairwise differences between outbreak isolates were compared. Within each outbreak, there were very few (i.e., ≤5) genomic differences across subtyping methods, except for an international travel-associated outbreak, where there were 19 SNP differences compared to 8 cgMLST allele differences. In this outbreak, hqSNP results were more closely aligned with wgMLST (chrom) results (16 allelic differences), suggesting that the additional allelic differences likely occurred on loci belonging to the accessory genome. This implies that wgMLST, due to its enhanced resolution, may be used routinely in conjunction with cgMLST for comparing outbreak isolate sequences associated with international travel. This finding should be acknowledged, as outbreak sequences are frequently shared and compared between international public health organizations [48]. A future analysis that examines additional international travel-associated outbreaks is needed to support this finding and would be beneficial for international public health groups that engage in genomic data sharing and comparison.

Previous evaluations of the congruence between WGS-based subtyping methods using pairwise genomic differences have yielded similar results to those of this study. For example, Pearce et al.’s comparison of cgMLST and SNP typing within a European *Salmonella* Enteritidis outbreak demonstrated that cgMLST analysis using the EnteroBase scheme was congruent with an original SNP-based analysis, wgMLST analysis, and epidemiological data [49]. We emphasize Pearce et al.’s statement that cgMLST can be readily implemented in laboratories that have access to web-based bioinformatics analysis tools, something now available within the PulseNet 2.0 system. Similarly, Simon et al.’s comparison of WGS-based approaches for investigating a foodborne outbreak caused by *Salmonella* Derby in Germany found that both SNP- and cgMLST-based methods proved to be highly suitable for reliable cluster generation [50]. While these two previous evaluations focused on an individual outbreak caused by one particular serotype and our study comprises multiple outbreaks caused by varying serotypes, the overall conclusion is consistent across studies.

In this study, the concordance between SNP- and allele-based methods was statistically evaluated using Pearson correlation coefficients and simple linear regression. These approaches confirmed a direct linear relationship between subtyping methods and quantified the strength of their concordance with Pearson correlation coefficients between 0.86 and 0.91; all correlation coefficients were supported by 95% confidence intervals. A similar investigation conducted by Blanc et al.also used linear regression and correlation coefficients to show that for *Pseudomonas aeruginosa*, core and whole-genome MLST approaches were as discriminatory as SNP-based approaches during outbreak investigations with correlation coefficients between 0.78 and 0.99 [51]. More relative to *Escherichia*, Bernaquez et al. ranked the discriminatory power of four WGS-based subtyping methods applied to a dataset of *Shigella sonnei* and *flexneri* outbreak isolates using linear regression [52]. While our study did not attempt to rank each subtyping method according to its discriminatory power, the overall findings were similar, in that in both studies, SNP- and allele-based methods were highly comparable for clustering epidemiologically related isolates.

In this investigation, while each of the three subtyping methods showed high overall concordance using linear regression, hqSNP was found to be slightly more discriminating than cgMLST on average, in that there were slightly lower cgMLST allele differences (0.432); CI: [0.426, 0.437] per one hqSNP allele difference observed in the slopes of the trend lines. This finding was foreseen, since SNP analysis can include non-core genes and intergenic regions, capturing a wider array of genetic differences than cgMLST, which is restricted to the core genome and because multiple changes in the same locus only count as one difference in MLST-based methods. The resolution between wgMLST (chrom) and hqSNP results was almost 100% analogous, where slopes showed 0.966; CI: [0.956, 0.975] wgMLST allelic differences for every one hqSNP difference. This outcome provides additional assurance that the wgMLST (chrom) scheme within the PulseNet 2.0 system captures approximately the same genetic variation as SNP-based approaches for closely related strains. Previous comparative analyses in which PulseNet outbreak data were used yielded similar findings [11,25,30]. In the present study, as expected, wgMLST (chrom) was found to provide enhanced resolution over cgMLST, where there were 1.914; CI: [1.895, 1.933] wgMLST allelic differences for every 1 cgMLST difference. Because wgMLST comprises a larger set of loci, it provides a broader view of genetic diversity and can be used to provide further resolution as needed when comparing outbreak isolates.

While SNP analysis is currently considered a gold standard genomic comparison method, the data analysis process for SNP analysis requires a certain level of expertise, is computationally intensive, and requires the selection of a reference genome [11,53]. Allele-based sequence typing presents a fitting substitute, particularly for large national surveillance networks, such as PulseNet USA, as a gene-by-gene method, such as MLST, provides a balance between resolution and computational efficiency [54,55]. Core genome schemes have been shown to be standardized and scalable for interlaboratory comparisons for enteric pathogens, as they offer a unified nomenclature that can facilitate communication and data sharing between public health entities [56]. Additionally, establishing sequence quality thresholds helps standardize results across different laboratories, making it easier to compare and aggregate data on a national or global scale. While cgMLST schemes generally include those loci present in the majority (95–100%) of isolates in a given group of bacteria [49,54,56], in a large national surveillance network, such as PulseNet USA, a lower threshold (85%) of core genes detected has been established to adjust for the variability that may be due to differences in technical replication across the network. Finally, by focusing on core genes, cgMLST may reduce the noise introduced by variations in non-core or accessory genomes, which may be less informative for distinguishing closely related strains. However, as noted in this study, some outbreaks, including those associated with international travel, may benefit from being compared using the added precision of wgMLST. Thus, while cgMLST is well-suited for long-term and routine surveillance, higher-resolution methods, such as wgMLST and hqSNP, can be used in the context of acute outbreaks, especially for pathogens with diverse accessory genomes. Higher-resolution methods may also be useful for studies exploring variation in mobile genetic elements or virulence/resistance genes not found in all strains.

In this study, tanglegrams were used as a visual tool to compare two hierarchical clusterings: cg/wgMLST (chrom) vs. hqSNP and cgMLST vs. wgMLST (chrom) of the same set of isolate sequences. The tanglegrams also provided a quantitative assessment of clustering similarity through the generation of BGI and CCC values. CCC values reflected greater concordance between methods than did BGI values (100% of outbreaks had CCC values > 0.88, regardless of which two subtyping methods were compared, whereas BGI values ranged between 0.35 to 0.99 depending on the outbreak and methods compared). This observation is likely due to differences in the intended purpose of these two measures, where the purpose of the BGI is to calculate the similarity between two clustering results, and the purpose of the CCC is to calculate how well the hierarchical clustering preserves the actual pairwise distances between isolate sequences. Nonetheless, the tanglegrams created similar tree topologies and allowed us to effectively visualize the relationships between clustering structures. A previous study by Zhang et al. also used tanglegrams to show that SNP, cgMLST, and wgMLST congruously separated porcine and environmental STEC O157:H7 isolates into various phylogenetic groups and revealed high CCC values between 0.995 and 0.996 across subtyping methods [57]. While the tanglegrams in Zhang et al.’s study demonstrated the effective clustering of STEC isolates by source type, our tanglegrams demonstrated the clustering of outbreak versus sporadic/non-outbreak isolates. Nevertheless, both studies illustrate how tanglegrams can be a useful visualization tool for comparing and clustering genomic data, particularly when they are accompanied by a statistical quantification of tree similarity. We note that many other genomic clustering visualization tools are available and could have added value to this study, for example GrapeTree, an interactive tree visualization program within EnteroBase, which can be used to create phylogenies using EnteroBase’s wg/cgMLT schemes [58].

As an external form of validation, we applied an unsupervised machine learning technique, K-means analysis, to show how outbreak and sporadic/non-outbreak isolate sequences were effectively partitioned outside of the PulseNet 2.0 system. An initial step in performing K-means analysis involves identifying the optimal number of clusters in a dataset based on a score, such as the Silhouette score (as was used in this study), or Elbow or Gap score [45]. For this study, we chose to apply the Silhouette method, obtaining a Silhouette score for each outbreak, since the Silhouette method does not require a training set to evaluate clustering performance and has the added advantage of identifying outliers in a dataset [45]. Our approach was successful, but we note that several other methods and metrics are available for evaluating clustering performance within K-means analysis and should be explored using PulseNet 2.0 data. For example, Coipan et al. examined the consensus between the Silhouette score and additional indices such as Dunn2 and McClain–Rao internal validation indices for clustering genomic data obtained using wg/cgMLST and SNP workflows [28]. Their results showed that while there were slight variations across indices based on the workflow, all metrics yielded the same optimal number of clusters and effectively separated outbreak isolates from non-outbreak isolates [28]. In our study, K-means analysis correctly designated outbreak and sporadic/non-outbreak isolates into the appropriate groups based on ground truth data for all but three outbreaks (outbreaks 04, 06, and 11). In these three outbreaks, only 1–2 isolates per outbreak were incorrectly assigned to the wrong group (with the exception of outbreak 06, where *all* sporadic isolates were incorrectly assigned to the outbreak group; however, this incorrect assignment only occurred when using hqSNP data). For these three outbreaks and in particular for outbreak 06, even though the average silhouette width was maximized at 2, there was enough diversity among the sporadic isolates that *three* (instead of two) K-groups could have reasonably been produced (Supplementary Table S5), possibly explaining why the K-means analysis forced the sporadic isolates into the incorrect group. Still, the overall results of this external validation show that K-means analysis can be used as a reliable proxy for clustering genomic data or at least as a supplementary validation technique.

This study has some limitations. First, we excluded all genes predominantly found on mobile genetic elements (phage and/or plasmid) and instead chose to limit our analysis to core and chromosomally located accessory genes. We chose to use the chromosomal-only wgMLST scheme instead of the full accessory genome approach because chromosomal genes are generally more conserved and present in all or most isolates, making comparisons more reliable and robust. Additionally, it is well-known that differences on plasmid loci and/or those found on other mobile genetic elements in the accessory genome may cause inflated genetic variation, making comparisons between subtyping methods challenging [52]; therefore, we chose to exclude these so as not to introduce unnecessary variation. However, the increased resolution provided by using all loci (including those in the accessory genome) may reveal more about the evolutionary history and genetic relatedness of different strains, which could have potential value for epidemiologic investigations, particularly for those involving travel. Second, we limited this analysis to STEC outbreaks due to the predominance STEC has over other *E. coli* pathotypes in PulseNet USA surveillance, wherein STEC makes up approximately 80% of all *E. coli* pathotypes (not including EIEC/*Shigella*) submitted to PulseNet USA. An expansion of this study could examine additional pathotypes to determine if the same congruence between subtyping workflows observed in this study exists across non-STEC pathotypes. We propose that an entirely separate study is warranted for EIEC/*Shigella*, given that cases occur through continuous person-to-person transmission, predominantly involving men who have sex with men (MSM), leading to long-term and recurrent outbreaks [59]. Undoubtedly, *Shigella*-specific transmission patterns, along with the prolonged genetic evolution of outbreaks, necessitates a unique assessment of existing WGS-based subtyping methods and cluster interpretation criteria [52,60]. Third, this study included ten foodborne outbreaks and one travel-associated outbreak, but there were no animal-contact-associated outbreaks included in this study. Given that zoonotic outbreaks may show increased variation based on cgMLST due to the evolution of strains between animal and human hosts [61], an expansion of this study could examine how this increased variation may affect concordance between allele- and SNP-based outputs and/or whether genomic differences between animal and human sources occur in areas other than the core genome. Finally, this study was limited to domestic outbreaks and does not incorporate extensive recent global data on STEC and other *E. coli*. Admittedly, surveying broader international data, including patterns associated with antimicrobial resistance and virulence gene distribution, could reveal noteworthy genetic relationships that suggest mutual sources or transmission routes across different regions, as seen in Bakleh et al.’s recent systematic review [62].

## 5. Conclusions

This study demonstrates that the allele schemes and allele calling workflow integrated within the PulseNet 2.0 system reliably cluster STEC outbreak isolates with the same epidemiologic concordance as hqSNP. Using multiple techniques and statistical measures including pairwise differences, linear regression models, and tanglegrams, this study confirms that the PulseNet 2.0 system can be used to detect STEC outbreaks caused by different serotypes and sources. Further evaluation using K-means analysis as an unsupervised machine learning approach objectively validated the results of this study, ensuring that the results are meaningful and reproducible. Overall, this study suggests the use of cgMLST as an ideal WGS-based analysis technique for routine STEC outbreak detection within large public health networks, due to its scalability and concordance with hqSNP, while wgMLST and hqSNP analyses can be used when further precision is needed for comparing outbreak isolates.

## Figures and Tables

**Table 1:** Summary of outbreaks included in the study.

Outbreak Number (Assigned in Study)	PulseNet Outbreak Code *	Serotype	No. of Outbreak Isolates (Sporadic Isolates) **	Confirmed Source	Range of Collection Dates
01	1601MLEXK-1	O121:H19	68 (5)	flour	2016-01-02 to 2016-09-07
02	1603VAEXH-1	O157:H7	9 (2)	raw milk	2016-03-07 to 2016-03-20
03	1608MIEC5-1	O5:H9	12 (4)	cheese served at restaurant	2016-03-14 to 2016-08-02
04	1912IAEXW-1	O103:H2	26 (3)	clover sprouts	2019-11-26 to 2019-12-23
05	1911MNEXH-1	O157:H7	18 (5)	frozen pizza crust	2019-10-10 to 2019-12-15
06	1909CAEXH-1	O157:H7	22 (6)	romaine lettuce	2019-07-14 to 2019-09-11
07	2206MLEXD-1	O111:H8	11 (4)	international travel	2022-05-24 to 2022-07-07
08	1905MLEXK-1	O121:H19	22 (4)	bison (ground)	2019-03-23 to 2019-08-12
09	1902MLEVC-1	O26:H11	21 (3)	flour	2018-12-28 to 2019-05-29
10	1808MLEVC-1	O26:H11	19 (2)	beef (ground)	2018-07-09 to 2018-09-04
11	1712MLEXH-1	O157:H7	23 (8)	leafy greens	2017-11-10 to 2017-12-14

* PulseNet outbreak codes are designated by the 2-digit year in which the outbreak was detected, 2-digit month in which the outbreak was detected, lab ID/state in which the outbreak was detected (“ML” = multi-state), and 3-digit serotype code, followed by the cluster number [8]. If multiple outbreaks meet the same criteria, then the cluster number is changed from 1 to 2, 2 to 3, etc. For example, 1601MLEXK-1 represents the 1st multi-state *E. coli* O121 outbreak detected in January 2016. 1601MLEXK-2 represents the 2nd multi-state *E. coli* O121 outbreak detected in January 2016, and so on. ** All sporadic isolates were matched to the outbreak by serotype and had collection dates within six months of the outbreak’s median collection date.

**Table 2:** Range of hqSNP- and allele-based pairwise genomic differences between outbreak isolates using PulseNet 2.0.

Outbreak Number Assigned in Study	Outbreak Code	hqSNP	cgMLST	wgMLST (Chrom)
01	1601MLEXK-1	0–2	0–2	0–5
02	1603VAEXH-1	0–1	0–0	0–2
03	1608MIEC5-1	0–3	0–3	0–4
04	1912IAEXW-1	0–7	0–2	0–3
05	1911MNEXH-1	0–2	0–2	0–4
06	1909CAEXH-1	0–7	0–3	0–7
07	2206MLEXD-1	0–19	0–8	0–16
08	1905MLEXK-1	0–2	0–3	0–5
09	1902MLEVC-1	0–2	0–1	0–3
10	1808MLEVC-1	0–1	0–1	0–1
11	1712MLEXH-1	0–5	0–2	0–5

**Table 3:** Summary table of metrics (regression analysis).

	Slope Equation; [95% CI for Slope]	R^2^	Pearson Correlation Coefficient; [95% CI]
cgMLST vs. hqSNP	y = 0.432x + 0.08; [0.426, 0.437]	0.75	0.86; [0.858, 0.870]
wgMLST (chrom) vs. hqSNP	y = 0.966x + 0.29; [0.956, 0.975]	0.82	0.91; [0.904, 0.911]
cgMLST vs. wgMLST (chrom)	y = 1.914x + 0.35; [1.895, 1.933]	0.81	0.90; [0.895, 0.903]

**Table 4:** Summary table of metrics (phylogenetic clustering analysis).

	Range of BGI Values Across Outbreaks	Range of CCC Values Across Outbreaks
cgMLST vs. hqSNP	0.413–0.987	0.981–1.00
wgMLST (chrom) vs. hqSNP	0.354–0.936	0.877–1.00
cgMLST vs. wgMLST (chrom)	0.686–0.964	0.979–1.00

**Table 5:** Summary table of metrics (K-means analysis).

	Range of Maximum Silhouette Scores at K = 2 Across Out Breaks	Range of Average Silhouette Widths for Outbreak Isolate Groups	Range of Average Silhouette Widths for Sporadic Isolate Groups
cgMLST	0.81–0.93	0.92–0.99	0.34–0.93
wgMLST (chrom)	0.81–0.97	0.89–0.99	0.32–0.92
hqSNP	0.87–0.99	0.87–0.99	0.35–0.91

## Data Availability

Raw sequence data files for the 297 isolates included in this study have been deposited in the National Center for Biotechnology (NCBI) Sequence Read Archive (SRA) under Bioproject PRJNA218110 (PulseNet *Escherichia coli* and *Shigella* genome sequencing). Supplementary Table S1 contains biosample and SRA accession numbers for the 297 isolates used in this study. Supplementary Table S2 contains biosample and SRA accession numbers for an additional 11 closed reference sequences used in hqSNP analysis.

## Supplementary Materials

The following supporting information can be downloaded at: https://www.mdpi.com/article/10.3390/microorganisms13061310/s1. Table S1: Isolate accession numbers; Table S2: List of accession numbers for additional closed reference sequences used in hqSNP (Lyve-SET) analysis; Table S3: Loci names for schema; Table S4: Baker’s Gamma Indices (BGI) and Cophenetic Correlation Coefficients (CCC) for outbreak tanglegrams; Table S5: Silhouette method graphs; Table S6: Average Silhouette widths from K-means analysis; Table S7: K-means dendrograms.

## Abbreviations

The following abbreviations are used in this manuscript:

STEC	Shiga-toxin-producing *Escherichia coli*
WGS	Whole-genome sequencing
cgMLST	Core genome multi-locus sequence typing
wgMLST	Whole-genome multi-locus sequence typing
wgMLST (chrom)	Whole-genome multi-locus sequence typing using chromosome-associated loci
BGI	Baker’s Gamma Index
CCC	Cophenetic Correlation Coefficient
ETEC	Enterotoxigenic *E. coli*
EPEC	Enteropathogenic *E. coli*
EAEC	Enteroaggregative *E. coli*
DAEC	Diffusely Adherent *E. coli*
EIEC	Enterinvasive *E. coli*
HUS	Hemolytic uremic syndrome
CDC	Centers for Disease Control and Prevention
APHL	Association of Public Health Laboratories
PFGE	Pulsed-field gel electrophoresis
NCBI	National Center for Biotechnology Information
SRA	Sequence Read Archive
CI	Confidence interval

## Appendix A

### Comparison of Pairwise Differences Obtained from BioNumerics v.7.6.3 vs. PulseNet 2.0

An ancillary objective of this study was to evaluate the concordance between PulseNet’s former data management system (BioNumerics v.7.6.3) and the PulseNet 2.0 system. Pairwise allelic differences among outbreak isolates were compared between systems and were found to be highly comparable. For cgMLST, allelic differences were 100% analogous between systems for outbreaks 01–09, but for outbreaks 10 and 11, there was one additional cgMLST difference observed using BioNumerics (Table A1). There was more variation between systems observed for wgMLST (chrom), where 8 outbreaks showed slightly different wgMLST (chrom) allele ranges, but this variation included no more than 4 allelic differences between systems. Outbreaks 01, 02, 04, 10, and 11 showed between one and four additional wgMLST (chrom) allelic differences using BioNumerics compared to PulseNet 2.0, and outbreaks 05, 07, and 08 showed between one and two additional wgMLST (chrom) allelic differences using PulseNet 2.0 compared to BioNumerics. For outbreaks 03, 06, and 09, wgMLST (chrom) allelic differences were 100% analogous between systems (Table A1). For outbreak and sporadic/non-outbreak isolates, cgMLST allele difference ranges were 100% analogous between systems for all outbreaks except outbreak 09 where there was one additional allele difference using BioNumerics (Table A2). The range of wgMLST (chrom) allele differences varied between systems for all outbreaks (Table A2).

Overall, pairwise allelic differences obtained from each system were highly concordant across multiple sets of outbreak isolates, particularly when using the cgMLST scheme. This finding suggests that allele difference outputs using the PulseNet 2.0 allele calling workflow can be interpreted similarly to those derived from BioNumerics, omitting the need to adjust STEC cluster detection thresholds following the transition to PulseNet 2.0.

**Table A1:** Range of hqSNP and allele-based pairwise genomic differences between outbreak isolates; BioNumerics v.7.6.3 vs. PulseNet 2.0. Red text indicates allele range discrepancies between systems.

Outbreak Number Assigned in Study	Outbreak Code	SNP Differences	cgMLST Differences (BioNumerics)	cgMLST Differences (PN 2.0)	wgMLST (chrom) Differences (BioNumerics)	wgMLST (Chrom) Differences (PN 2.0)
01	1601MLEXK-1	0–2	0–2	0–2	0–6	0–5
02	1603VAEXH-1	0–1	0–0	0–0	0–4	0–2
03	1608MIEC5-1	0–3	0–3	0–3	0–4	0–4
04	1912IAEXW-1	0–7	0–2	0–2	0–4	0–3
05	1911MNEXH-1	0–2	0–2	0–2	0–2	0–4
06	1909CAEXH-1	0–7	0–3	0–3	0–7	0–7
07	2206MLEXD-1	0–19	0–8	0–8	0–15	0–16
08	1905MLEXK-1	0–2	0–3	0–3	0–4	0–5
09	1902MLEVC-1	0–2	0–1	0–1	0–3	0–3
10	1808MLEVC-1	0–1	0–2	0–1	0–5	0–1
11	1712MLEXH-1	0–5	0–3	0–2	0–7	0–5

**Table A2:** Range of hqSNP and allele-based pairwise genomic differences between outbreak + sporadic/non-outbreak isolates; BioNumerics v.7.6.3 vs. PulseNet 2.0. Red text indicates allele range discrepancies between systems.

Outbreak Number Assigned in Study	Outbreak Code	SNP Differences	cgMLST Differences (BioNumerics)	cgMLST Differences (PN 2.0)	wgMLST (Chrom) Differences (BioNumerics)	wgMLST (Chrom) Differences (PN 2.0)
01	1601MLEXK-1	0–40	0–21	0–21	0–47	0–39
02	1603VAEXH-1	0–63	0–26	0–26	0–70	0–59
03	1608MIEC5-1	0–106	0–34	0–34	0–83	0–70
04	1912IAEXW-1	0–122	0–61	0–61	0–127	0–105
05	1911MNEXH-1	0–182	0–88	0–88	0–171	0–158
06	1909CAEXH-1	0–150	0–27	0–27	0–60	0–59
07	2206MLEXD-1	0–114	0–54	0–54	0–111	0–100
08	1905MLEXK-1	0–112	0–45	0–45	0–91	0–84
09	1902MLEVC-1	0–85	0–36	0–35	0–103	0–92
10	1808MLEVC-1	0–53	0–27	0–27	0–53	0–44
11	1712MLEXH-1	0–49	0–20	0–20	0–48	0–44
